# Memory and truth: correcting errors with true feedback versus overwriting correct answers with errors

**DOI:** 10.1186/s41235-019-0153-8

**Published:** 2019-02-13

**Authors:** Janet Metcalfe, Teal S. Eich

**Affiliations:** 1 Department of Psychology, Columbia University, New York, USA; 2 Department of Neurology, Columbia University, New York, USA

## Abstract

In five experiments, we examined the conditions under which participants remembered true and false information given as feedback. Participants answered general information questions, expressed their confidence in the correctness of their answers, and were given true or false feedback. In all five experiments, participants hypercorrected when they had made a mistake; that is, they remembered better the correct feedback to errors made with high compared to low confidence. However, in none of the experiments did participants hyper'correct' when false feedback followed an initially correct response. Telling people whether the feedback was right or wrong made little difference, suggesting that people already knew whether the feedback was true or false and differentially encoded the true feedback compared to the false feedback. An exception occurred when false feedback followed an error: participants hyper'corrected' to this false feedback, suggesting that when people are wrong initially, they are susceptible to further incorrect information. These results indicate that people have some kind of privileged access to whether their answers are right or wrong, above and beyond their confidence ratings, and that they behave differently when trying to remember new “corrective” information depending upon whether they, themselves, were right or wrong initially. The likely source of this additional information is knowledge about the truth of the feedback, which they rapidly process and use to modulate memory encoding.

**Electronic supplementary material:**

The online version of this article (10.1186/s41235-019-0153-8) contains supplementary material, which is available to authorized users.

## Significance

This research addresses the issue of how people update both correct and incorrect semantic information, and whether their confidence in the truth of their initial responses impacts that updating. When people are given corrective feedback after having committed a high-confidence error, they are more likely to remember that feedback than when given feedback after having committed low-confidence errors. It is shown here that this hyper'correction'^1^ of high-confidence errors occurs regardless of whether the feedback is true or false. However, when they are given false feedback after having produced a high- versus low-confidence correct response, such hypercorrection does not occur. Two conclusions emerge. First, people have some knowledge of the truth of their answer above and beyond their stated confidence in its truth when they produce the answer. It is likely that this additional knowledge stems from an evaluation of the truth of the feedback. Second, people are vulnerable to false information when they have made a high-confidence error. In this case, they appear to be unable to evaluate the truth of the feedback, and hence, hyper-encode the true and false information equivalently.

## Background

While the concept of deliberately or intentionally conveying false information dates back hundreds of years (e.g., Octavian’s and Mark Anthony’s propaganda war dating to ~33 BCE), what causes an individual to be susceptible to false information or to believe true compared to false information has yet to be identified. Many studies have shown that an individual’s confidence moderates their ability to update misinformation with true information. When an individual generates an incorrect answer to a question and is then provided with the correct answer, they are considerably more likely to remember the correct answer when the initial error was made with high rather than low confidence (Butterfield & Mangels, 2003; Butterfield & Metcalfe, 2001, 2006; Fazio & Marsh, 2009, 2010; Huelser & Metcalfe, 2012; Kang et al., 2011; Kulhavy, Yekovich, & Dyer, 1976; Metcalfe, Butterfield, Habeck, & Stern, 2012; Metcalfe & Finn, 2012; Sitzman, Rhodes, & Tauber, 2014). This enhanced memory for corrective feedback to high-confidence errors is called the hypercorrection effect. It has been shown with children (Metcalfe & Finn, 2011), young adults (Butterfield & Metcalfe, 2006, and see Metcalfe, 2017, for review), and—to a lesser extent—older adults (Cyr & Anderson, 2013; Eich, Stern, & Metcalfe, 2013; Metcalfe, Casal-Roscum, Radin, & Friedman, 2015; Sitzman, Rhodes, Tauber, & Liceralde, 2015). The hypercorrection effect occurs with both immediate and delayed testing (Butterfield & Mangels, 2003; Metcalfe & Miele, 2014), and it is found with general information questions as well as with conceptual inferences, classroom materials, and lexical memory (Iwaki, Matsushima, & Kodaira, 2013; van Loon, Dunlosky, van Gog, van Merrienboer, & de Bruin, 2015).

Despite converging evidence for the effect of confidence on later memory when an error was made initially, in all previous experiments of the hypercorrection effect, the feedback given to participants has always been factually correct. Thus, the question remains of whether hyper'correction' would occur for false feedback given to an initially correct response. This question is relevant to many domains. For example, in the classroom, a student may hear another student’s incorrect response to a question. Likewise, “alternative facts” have become commonplace in today’s parlance. Under what conditions are people likely to adopt misinformation and incorporate it into their belief system?

Several theoretical positions concerning human memory suggest that when people generate an answer that they assert with confidence to be true, they have no knowledge over and above those feelings of confidence of whether their answer is, in actuality, true or not. However, there is always the possibility that the ratings are made impulsively and could be made more accurately upon further deliberation (e.g., Buratti, Allwood, & Kleitman, 2013; Koriat, Lichtenstein, & Fischhoff, 1980). However, aside from the possibility that there might be some latent knowledge that the person could theoretically tap into when making a judgment, stated confidence is taken to be a straightforward reflection of subjective knowledge. If true and false retrievals from memory are not distinguishable, and our evaluation of or confidence in the correctness of our answers is based on heuristics that should apply equally for right and wrong retrievals, then the correction of errors given true feedback may be indistinguishable from the updating of correct answers by false feedback. If this is the case, then individuals may show the same confidence-related hyper'correction' to feedback when they had been correct just as they do when they were incorrect. On the other hand, while confidence may be highly associated with prior knowledge, other factors may could influence how a person responds to feedback and updates memory. For example, if they have some additional knowledge beyond their stated confidence, or if underlying memory structures are different for correct vs. incorrect responses or for true or false feedback, then they might show a different pattern of confidence-related memory updating. It is this basic idea that we test in the five experiments that follow. We investigate this question through the lens of the so-called hypercorrection effect, a phenomenon that is characteristic of the correction of high-confidence errors.

In support of the conjecture that people have no way, over and above their confidence judgments, to distinguish between whether they have generated the right answer or an error, Koriat’s (1993, 2008, 2011, 2012, 2018, and see Koriat & Goldsmith, 1996) accessibility/consensuality model proposes that metacognitive confidence judgments—indicating people’s certainty about the correctness of the answers they retrieve from memory—are based on the mnemonic cues of self-consistency, accessibility, familiarity, and fluency, even though they may hardly be aware of relying on these cues. Koriat (2008) notes: “It might be futile to expect that metacognitive judgments such as feeling of knowing or subjective confidence would have privileged access to the correct target” (p. 954). Although Koriat acknowledges that high confidence is usually associated with correct responses, he argues that this typically found correlation does not occur because people have privileged access to the correctness of their answers. Rather, it occurs because, generally, people access correct rather than incorrect information from memory. However, he argues that when the wrong response is the consensual response, it, too, will be endorsed with high confidence. In that case, metacognitive judgments will be counter-diagnostic of the correctness of the answer (see, Koriat, 1993, 2018).

Similarly, Lewandowsky, Ecker, Seifert, Schwarz, and Cook (2012) propose that many factors, including ideology and personal world views, influence people’s overt evaluation of memorial information, as well as the likelihood that they will update misinformation when provided with correct information. They note that much of the information that people use to make assessments of the validity of information (Ecker, Lewandowsky, & Apai, 2011) is not necessarily diagnostic of correctness. Considering only the basic memory processes themselves, though, they suggest that there should be no fundamental cognitive difference between correcting errors when given the right answer and overwriting correct information with wrong answers: “Correcting misinformation is cognitively indistinguishable from misinforming people to replace their preexisting correct beliefs” (Lewandowsky et al., 2012, p. 124). Indeed, they argue that this purported indifference to the actual truth of responses may be responsible, in part, for why misinformation is so pernicious.

Finally, when people produce an answer, even if they are unable to tell—over and above what is indicated by their confidence—whether that answer is correct or incorrect, they may, nevertheless, be able to engage additional cognitive processes when feedback is given. They may think that they are correct when they generate a high-confidence answer, but upon being given discrepant feedback realize—because of that additional information—that they had been wrong. Conversely, if they were correct but given false feedback, the feedback itself may provide additional information that solidifies the initially correct response—allowing them to resist the memory meddling that might accrue to false feedback. Indeed, Rich, Van Loon, Dunlosky, and Zaragoza (2017) recently found that belief in the feedback modulates the correction of errors. Experiments 2 and 5 of the present series of experiments directly address the possibility that the feedback itself may be sufficient—at least in some cases—to allow people to ascertain whether their answers were in fact correct or not, and to update their memories differentially as a result of its truth value.

The first question that we address, then, is whether people selectively remember feedback given to *all* high- compared to low-confidence responses, or whether, instead, they only hypercorrect when they were wrong initially. The second question, which we investigate selectively following the commission of erroneous responses, is whether the hypercorrection effect occurs regardless of whether the feedback given to errors is true or false. The third question we address relates to the memorial role of the possible recognition of the truth value of the feedback. If people respond differently when they are wrong as opposed to when they are right, it is still possible—as is consistent with the no-privileged-access view—that they did not know whether their answers were right or wrong, over and above their stated confidence, but they obtain further information from the feedback that allows them to modify their memory encoding.

To investigate these issues, in a series of five experiments modeled on the basic hypercorrection paradigm (Butterfield & Metcalfe, 2001), we asked people to produce answers to general information questions and to provide their confidence in their answers. Unlike other studies of the hypercorrection effect in which the feedback was always the correct response, the feedback provided following confidence ratings in the current set of experiments could be either true or false. In the first two experiments, participants were provided with true feedback when they made an error on the initial question, and false (but highly plausible) feedback when they were correct. In Experiments 3 and 4, for initially correct responses, true feedback was provided half of the time, and the other half of the time participants were asked to remember false feedback. If people hypercorrect all high confidence answers--regardless of truth-value-- then all conditions should show the hypercorrection effect. For all experiments, then, memory for the feedback (whether true or false), as a function of people's confidence in the correctness of their original answers, as well as their knowledge of the truth-value of the feedback, was investigated. 

## General method

### Design

All five experiments were divided into three phases. In Phase 1, each participant was asked a series of randomly ordered general information questions. For each question, participants typed in their answer, and then indicated their confidence in its correctness on a slider scale ranging from “sure wrong” on the far left to “sure correct” on the far right, corresponding to values ranging from 0 to 100. Then, with the question, but not the participant’s answer, still on screen, feedback, consisting of a to-be-learned word in red, was presented for 2 s. This sequence was repeated until all items had been presented.

In Phase 2, each question was re-presented, in a random order. Participants were asked to type in the response to that question that had been presented in red during Phase 1 (i.e., the feedback). They were then asked to provide their confidence concerning whether they had correctly typed in the word that had been presented in red.

Finally, in Phase 3, the participants were presented with all the questions again and were asked to provide the factually correct answer to each question, along with their confidence in the truth value of their answer. After the participants in Phase 3 provided what they believed was the correct answer, they were then shown the factually correct answer along with the words: “The correct answer is: _____.” This final, corrective feedback to all questions was included to ensure that participants left the experiments having been told, unambiguously, the correct answers to all questions.

To evaluate whether participants hypercorrected in each of the experiments under different conditions, gamma (γ) correlations (Goodman & Kruskal, 1954) were computed between the confidence on the initial responses, and whether, in Phase 2, the word that was given in red to be learned was recalled.^2^ The γ correlation between confidence in the original response and responding correctly in the final, Phase 3 test (when the participant was asked to give the correct answer, and not necessarily the answer that was given in red as feedback) was also computed for all experiments. The criterion for hypercorrection was a γ correlation significantly greater than zero. Note that “correct” in Phases 1 and 3 reflects factual accuracy, whereas “correct” in Phase 2 reflects recall of the feedback word printed in red, regardless of its factual accuracy.

### Materials

The general information questions (see Butterfield & Metcalfe, 2006) used in the current experiments included questions from Nelson and Narens (1980), various board games, and internet trivia sites. A latent semantic analysis (LSA; Landauer & Dumais, 1997; Landauer, Foltz, & Laham, 1998) of the relation of the question to the correct answer, and the question to the designated wrong answer, was conducted on the core set of 64 questions (given in Additional filr 1; additional questions were added for Experiment 4 only, to allow replication and to provide more data in each within-participant condition). The average LSA values^3^ were well above zero (which would indicate no relation): *M* = .212, standard deviation (*SD*) = .209, *t*(63) = 8.10, *p* < .001, between the questions and the correct answers, and *M* = *.*248, *SD* = .14, *t*(62) = 13.56, *p* < .001, between the question and the incorrect answers. A paired *t*-test using question (instead of participant) in the analysis revealed no significant difference (*M* difference = −.03) between the LSA values of the correct answers’ and the incorrect answers’ relation to the question (*t*(59) = 1.55, *p* = .125). As these analyses show, the incorrect answers that were designated a priori to be learned, in these experiments, were as closely associated with the questions as were the correct answers. The only discernible difference was in the truth value of the answers.

### Procedure

Participants in all five experiments were tested individually on iMac computers programmed in RealStudio (now called Xojo). They were instructed that they would be reading a list of general information questions and that after each question they should type in the correct answer. Participants were told not to make spelling mistakes. Spelling errors were manually checked and were rare. They were told that they would then be shown by the computer, in red, an answer that might be different from the answer that they had provided. Their job was to learn the answer in red for a later test in which they would be shown the question and would be asked to give the word that had been presented in red. All procedures used in this series of experiments conformed to Psychonomic Society ethical guidelines and were approved by the Columbia University Institutional Review Board.

## Experiment 1

The first experiment investigated recall of the feedback provided following high-confidence compared to low-confidence original responses under two different conditions: (1) when true feedback followed an initial error and (2) when false feedback followed an answer that was initially correct. The first situation has been extensively investigated and is the condition under which the standard hypercorrection effect is found. The second situation has not previously been investigated, within the hypercorrection paradigm. The hypothesis—gleaned from the no-privileged-access view—was that hypercorrection of high-confidence responses would occur both when people had been wrong and were given the right answers, and when they had initially been right and were given wrong answers.

### Methods

In Phase 1, participants were asked 64 randomly ordered general information questions. If a participant’s answer was correct, they were given feedback in red that was incorrect but plausible (see Materials section). If the participant’s answer was wrong, the feedback in red was the correct answer. Participants were not told whether the to-be-learned item in red was true (i.e., factually correct) or false (i.e., factually incorrect). In Phase 2 of the experiment, the 64 questions were re-presented in a random order. Participants were asked to type in the response to a question that had been presented in red and to provide their confidence concerning whether they had correctly typed in the word that had been presented in red. Finally, in Phase 3, the participants were presented with all the questions again and were asked to provide the factually correct answer to each question, along with their confidence in their answer. They were then shown the factually correct answer. Participants were 29 Columbia University students (8 male and 18 female, plus 3 who did not answer, with an average age of 21.43 years) who engaged in the experiment in exchange for a course credit. All except three participants also wrote short essays for their class about the experiment.

### Results

#### Phase 2

##### Hypercorrection

**Fig. 1:**
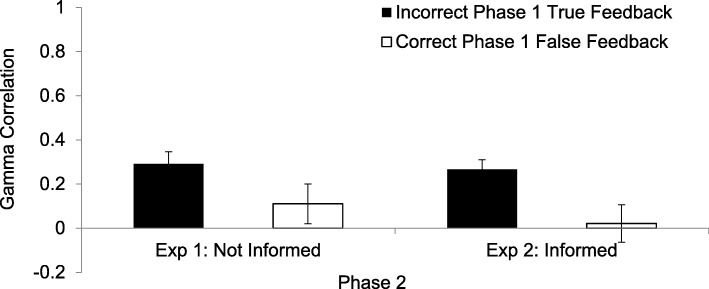
The γ correlations between confidence in the factual accuracy of responses in the initial test and correct performance in Phase 2, in which recalling the red feedback word, regardless of whether it was true or false, was considered correct. The solid black bar represents the standard hypercorrection condition in which the participant answered incorrectly in Phase 1 and was given true feedback. The white bar provides the γ correlations when the participant responded correctly in Phase 1 and false feedback was given. On the left are the results from Experiment 1, in which participants were not informed about whether the to-be-learned word, provided as feedback in red, was true or false. On the right are the results from Experiment 2, in which participants were informed, immediately upon being presented with the to-be-learned word in red, whether the feedback was true or false. Error bars indicate the standard error of the mean

As can be seen on the left of Fig. 1, when participants had produced erroneous responses and were given the correct answer, a hypercorrection effect was found such that the γ correlation (*M* γ = *.*29, *SD* = .29) between initial confidence and recall of the factually correct red response was significantly greater than zero (*t*(28) = 5.26, *p* < .001). When, however, they had been correct initially and were given false feedback to recall, participants did not hyper'correct' (*M* γ = *.*11, *SD* = .47, *t*(26) = 1.22, *p* = .232). Although the former correlation differed from zero whereas the latter did not, the two γ correlations were not significantly different from one another (*t*(26) = 1.60, *p* = .120).

##### Recall

The recall of the to-be-learned responses in red was significantly higher when the original answer had been correct (*M* = *.*68, *SD* = .20), compared to when the original answer had been incorrect (*M* = .58, *SD* = .19, *t*(28) = 2.31, *p* = .028), indicating that the incorrect answers given following correct responses were remembered better than were the correct responses given as feedback to errors. All recall means are presented in Table 1.

**Table 1:** Proportion of correct recall by phase depending on whether the given answer in Phase 1 was correct or incorrect and whether the feedback given to the response was true or false

Phase	Phase 1 response	Feedback	Experiment				
			1	2	3	4	5
2	Incorrect	True	.58 (.19)	.67 (.15)	.65 (.15)	.53 (.15)	.56 (.24)
		False			.62 (.16)	.54 (.17)	.43 (.19)
	Correct	False	.68 (.20)	.58 (.21)	.70 (.30)	.71 (.21)	.56 (.24)
		True			.99 (.03)	.96 (.21)	.95 (.15)
3	Incorrect	True	.48 (.20)	.66 (.16)	.56 (.14)	.42 (14)	.63 (.17)
		False			.02 (.03)	.03 (.03)	.55 (.20)
	Correct	False	.60 (.31)	.85 (.24)	.69 (.31)	.87 (.22)	.99 (.05)
		True			.99 (.03)	.95 (.12)	.97 (.07)

#### Phase 3

##### Relation of Phase 1 confidence to Phase 3 factual recall

**Fig. 2:**
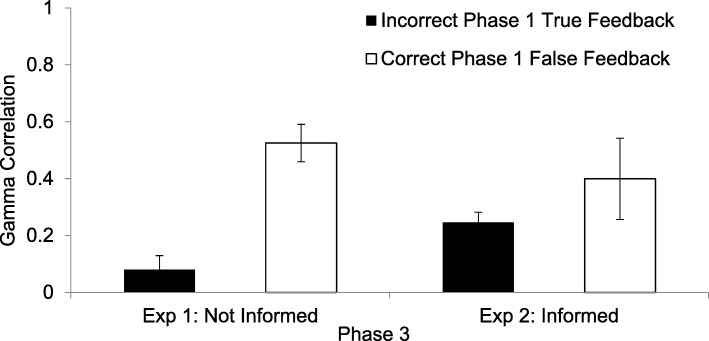
The γ correlations between confidence in the factual accuracy of responses in the initial test and correct performance in Phase 3, in which participants were retested and were asked to provide the factually correct answer to each question. The solid black bar represents the standard hypercorrection condition in which an error had been committed in Phase 1 and true feedback was given. The white bar represents the condition in which the participant had originally been correct in Phase 1 and false feedback was given. On the left are results from Experiment 1, in which participants were not informed about whether the to-be-learned word, provided as feedback in red, was true or false. On the right are results from Experiment 2, in which participants were informed, immediately upon being presented with the to-be-learned word in red, whether the feedback was true or false. Error bars indicate the standard error of the mean

As can be seen on the left of Fig. 2, the γ correlations between confidence in the original response and factually correct responding in the Phase 3 test were significantly positive for answers that were correct initially but for which false feedback was given, indicating that participants reverted to their originally correct answers on the final test, especially when they had been highly confident originally (*M* γ = .52, *SD* = .33, *t*(25) = 8.01, *p* < .001). However, even though they had been given the correct answers as feedback to their erroneous responses, participants in this experiment did not significantly hypercorrect in Phase 3. The mean γ correlation was only .08 (*SD* = .27), which was not significantly different from zero (*t*(28) = 1.63, *p* = .115). The Phase 3 γ correlations in the two conditions were significantly different from one another (*t*(25) = −5.38, *p* < .001).

##### Recall

As shown in Table 1, participants in Phase 3 gave the factually correct response marginally more frequently when they had been correct originally but had been given false feedback (*M* = .60, *SD* = .31) than when they had been wrong originally but had been given correct feedback (*M* = .48, *SD* = .20, *t*(28) = 1.99, *p* = .057). Performance in Phase 3, where participants were asked to provide the factually correct answers to the questions, declined by 40% for items for which participants had been correctly initially, as a function of false feedback.

### Discussion

The results of Experiment 1 indicate that participants hypercorrected on the immediate test when they were given correct feedback to their own errors. The results also reveal that participants did not hyper'correct' on the immediate test when they had made a correct response with high confidence but were then given *false* feedback. This finding weighs against the no-privileged-access view and is contrary to the hypothesis that people cannot distinguish true from false information and will, therefore, hypercorrect solely as a function of their confidence.

Furthermore, although a hypercorrection effect was found in the standard condition with the immediate test, this effect was not significantly different from zero on the retest after a slight delay. Several previous studies have shown that the standard hypercorrection effect persists after a delay of a week or more under conditions in which the factually correct answer is provided (Butterfield & Mangels, 2003; Metcalfe & Miele, 2014). It appears that the uncertainty introduced by including some questions for which incorrect feedback followed correct answers may have undermined the stability of the hypercorrection effect.

## Experiment 2

The results of Experiment 1 suggest that people may have some knowledge, other than their stated confidence ratings, about whether their answers were right or wrong that was likely responsible for the differential memory for high-confidence error feedback compared to high-confidence correct feedback. It seems likely that people quickly assessed the truth of the feedback and used this truth value to modulate their memory encoding of the to-be-remembered word in red. If there was such a modulation, it likely occurred because people knew, without anyone telling them, whether the feedback was true or false. Accordingly, Experiment 2 was identical to Experiment 1, except that after each to-be-learned red feedback word was presented in Phase 1, a message box told the participants, explicitly, whether the red word was the factually correct answer or not. Participants were also told, at the outset of the experiment, that when they got an answer correct, the computer would present an incorrect alternative for them to learn, and when they got the answer wrong, it would present the correct alternative for them to learn. Participants were 29 Columbia University students (18 female and 10 male, plus 1 who did not answer, with an average age of 19.7 years).

### Results

#### Phase 2

##### Hypercorrection

As can be seen on the right of Fig. 1, participants hypercorrected when they were given the correct answers following an error (*M* γ = .27, *SD* = .23, *t*(27) = 6.19, *p* < .001). However, they showed no hyper'correction' when their original responses had been correct and they were asked to recall false feedback in Phase 2 (*M* γ = .02, *SD* = .44, *t*(26) = .25, *p* = .800). The difference between these two conditions was significant (*t*(26) = 2.58, *p* = .016).

##### Recall

There was a marginal difference between recall of the to-be-learned responses in red when the original answer had been correct (and thus, false feedback was to be recalled; *M* = *.*58, *SD* = .21), compared to when the original answer had been incorrect (and thus, true feedback was to be recalled; *M* = .67, *SD* = .15, *t*(27) = − 1.84, *p* = .078), such that the recollection of the true feedback following an error was higher than the recall of false feedback following an initially correct response.

#### Phase 3

##### Relation of Phase 1 confidence to Phase 3 factual recall

As shown on the right of Fig. 2, the hypercorrection effect, in the standard condition, persisted strongly in Phase 3 (*M* γ = *.*25, *SD* = .19, *t*(27) = 6.93, *p* < .001). When participants had initially given the correct answer, were told that they were correct, but were then given false feedback to remember, they were more likely to correctly produce high-confidence than low-confidence (initially correct) responses in Phase 3 (*M* γ = *.*40, *SD* = .49, *t*(11) = 2.79, *p* = .017).

##### Recall

Participants in Phase 3 gave the factually correct responses more frequently when they had been correct originally but given false feedback (*M* = .85, *SD* = .24) than when they had been wrong originally but had been given correct feedback (*M* = .66, *SD* = .16, *t*(27) = 6.30, *p* < .000).

## Comparison of Experiments 1 and 2

### Phase 2

#### Hypercorrection

To examine the effect of uncertainty about whether the feedback was true or false, the γ correlations that are relevant to hyper'correcting' from Experiment 1 were compared to those of Experiment 2. A 2 × 2 analysis of variance (ANOVA), treating experiment as a between-participant independent variable and true or false feedback following their initial response as a within-participant variable, showed no effect of experiment (*F*(1, 52) < 1). There was a main effect of condition (*F*(1, 52) = 8.53, *p* = .005, ${\eta}_p^2$ = .14, 1 – β = .82), such that there was a greater hypercorrection effect to high-confidence responses following errors (*M* = .27, *SD* = .27) than following correct responses (*M* = .07, *SD* = .45). There was no significant interaction between experiment and condition (*F*(1, 52) < 1). Thus, the pattern of results across both experiments was equivalent. Participants’ hypercorrection performance for Phase 2 was the same whether they were given explicit experimenter-provided information about the correctness of the feedback or not.

#### Recall

Correct recall of the word in red in Phase 2 did not differ as a function of either condition (false feedback following a correct response vs. true feedback following an incorrect response) or experiment, both *F*s(1, 55) < 1. There was an interaction between condition and experiment (*F*(1, 55) = 8.53, *p* = .005,${\eta}_p^2$ = .13, 1 – β = .82), such that being correct in Phase 1 and receiving false feedback resulted in marginally significantly better performance in Experiment 1 (*M* = *.*68, *SD* = .20) than in Experiment 2 (*M* = .58, *SD* = .21, *t*(55) = 1.908, *p* = .06). On the other hand, being wrong in Phase 1 and receiving true feedback resulted in marginally significantly better performance on Phase 2 in Experiment 2 (*M* = .67, *SD* = .15) than in Experiment 1 (*M* = .58, *SD* = .19, *t*(55) = 1.84, *p* = .07).

### Phase 3

#### Relation of Phase 1 confidence to Phase 3 factual recall

There was no overall effect of experiment (*F* < 1). There was a main effect of condition (*F*(1, 52) = 8.53, *p* = .005, ${\eta}_p^2$ = .14, 1 – β = .82), such that there was a greater hypercorrection effect to high-confidence responses following correct initial responses (*M* = .49, *SD* = .39) than following initial errors (*M* = .10, *SD* = .23). There was, however, no interaction between experiment and condition *(F*(1, 36) = 2.04, *p* = .16).

#### Recall

There was an overall effect of experiment such that recall of the factually correct answer in Phase 3 was higher in Experiment 2 (*M* = *.*76, *SD* = .04) than in Experiment 1 (*M* = .54, *SD* = .04, *F*(1, 55) = 17.17, *p* < .001, ${\eta}_p^2$ = .24, 1 – β = .98). There was a main effect of condition such that recall was higher when participants had been correct initially and given false feedback (*M* = .73, *SD* = .04) than when they had been wrong and provided with correct feedback (*M* = .57, *SD* = .02, *F*(1, 55) = 20.43, *p* < .001, ${\eta}_p^2$ = .27, 1 – β = .99). There was no interaction between experiment and condition (*F*(1, 55) < 1).

### Discussion

Together, the results of Experiments 1 and 2 suggest that increasing people’s certainty about the correctness of the feedback had little impact on hypercorrection and recall performance, as if participants knew whether they were right or wrong to begin with. There was no difference in the standard hypercorrection effect or in the lack of a hyper'correction' effect when people were correct and given false feedback. There was, however, a difference in the persistence of the standard hypercorrection effect after a delay. In Experiment 1, in which they were uncertain, the delayed hypercorrection effect was smaller than in Experiment 2, in which there was no uncertainty. Further, people’s factually correct performance on the final test was different depending on whether the participants were explicitly told that the false feedback had been incorrect.

## Experiment 3

In the first two experiments, the truth or falsity of the feedback was perfectly negatively correlated with whether the participant’s initial answer had been right or wrong. Given that the data indicate that people responded differently to questions on which they had been correct and incorrect, this correlation may have been problematic. It is possible, for instance, that participants might have noticed it and used this information to alter their learning strategies. Accordingly, in the last three experiments, the truth or falsity of the feedback was randomly assigned with respect to the correctness of participants’ initial responses.

### Method

In Experiment 3 there were four conditions: (1) incorrect followed by true feedback, (2) incorrect followed by false feedback, (3) correct followed by false feedback, and (4) correct followed by true feedback. As in the first two experiments, participants were retested in Phase 3 for the factually correct answers, made a confidence rating about their response, and then were told the factually correct answer for each question. Participants were 24 Columbia University students (12 female and 12 male, with an average age of 20.25 years). The participants received a bonus course credit for participating in the experiment.

### Results

#### Phase 2

##### Hypercorrection^4^

**Fig. 3:**
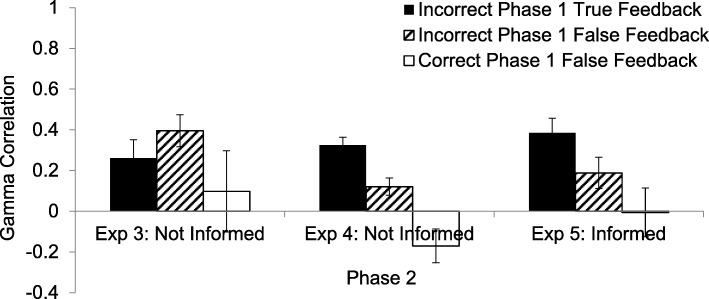
The γ correlations between confidence in the factual accuracy of responses on the initial, Phase 1 test and correct performance in Phase 2, in which recalling the feedback word, regardless of whether it was true or false, was considered correct. The solid black bar represents the standard hypercorrection condition in which the participant answered incorrectly in Phase 1 and was given true feedback. The hatched bar represents the condition in which the participant answered incorrectly in Phase 1 and was given false feedback. The white bar indicates the condition in which the participant answered correctly in Phase 1 and was given false feedback. When people were correct in Phase 1 and then were given correct feedback, they were, of course, virtually always correct, making it impossible to compute gammas in this condition. So, this condition is not included in this graph. In the left and center are the results from Experiments 3 and 4, respectively, in which participants were not informed about whether the to-be-learned word, provided as feedback, was true or false. On the right are the results from Experiment 5 in which participants were informed, immediately upon being presented with the to-be-learned word in red, whether that feedback was true or false, and, if false, what the true answer was. Error bars indicate the standard error of the mean

As is shown in the left of Fig. 3, a hypercorrection effect was observed in the standard condition in which participants made an error and then received true feedback (*M* γ = .26, *SD* = .44, *t*(23) = 2.91, *p* = .008). There was also a hyper'correction' effect when participants had made an initial error and *false* feedback was given to them (*M* γ = .40, *SD* = .38, *t*(23) = 5.04, *p* < .001). To assess whether this effect stemmed from participants receiving as false feedback the same, erroneous answer that they had given as their response in Phase 1,^5^ a second hyper'correction' γ was computed in which all trials in which the original error was the same as the to-be-learned (false) feedback in Phase 2 were removed. The resulting γ correlation was still significantly positive (*M* γ = .41, *t*(23) = 4.40, *p* < .001). In the condition in which the initial response was correct and false feedback was given, the γ correlation between original confidence and production of the response in red was not significantly different from zero (*M* = .09, *SD* = .80, *t*(15) = .49, *p* = .630), indicating that participants in this condition did not hyper'correct'. The difference among the three conditions was not significant in this experiment (*F*(2, 30) < 1, ${\eta}_p^2$ = .05, 1 – β = .16), possibly because many participants did not provide observations in all three conditions, and the power was low.

##### Recall

As indicated in Table 1, when participants were correct in Phase 1 and were given true feedback, they were virtually always correct in Phase 2 (*M* = .99, *SD* = .03). A repeated measures ANOVA was conducted on the remaining three conditions (correct in Phase 1 followed by false feedback, incorrect in Phase 1 followed by true feedback, and incorrect in Phase 1 followed by false feedback). The means, respectively, were: *M* = .70, *SD* = .30; *M* = .65, *SD* = .15; and *M* = .62, *SD* = .16. The main effect of condition was not significant (*F*(2, 46) = 1.65, *p* = .204, $, {\eta}_p^2$ = .07, 1 – β = .33).

#### Phase 3

##### Relation of Phase 1 confidence to Phase 3 factual recall

**Fig. 4:**
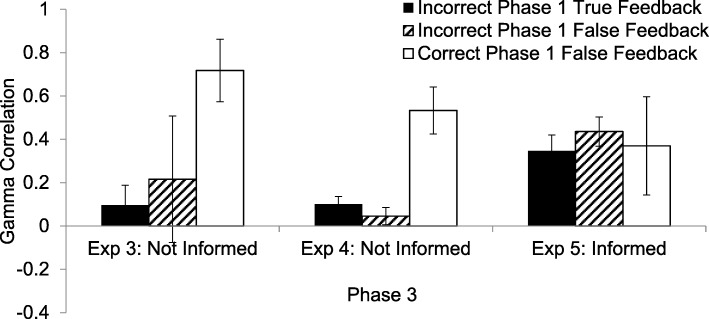
The γ correlations between confidence in the factual accuracy of responses on the initial, Phase 1 test and correct performance in Phase 3, in which participants were retested and were asked to provide the factually correct answer to each question. The solid black bar represents the standard hypercorrection condition in which the participant answered incorrectly in Phase 1 and was given true feedback. The hatched bar represents the condition in which the participant answered incorrectly in Phase 1 and was given false feedback. The white bar indicates the condition in which the participant answered correctly in Phase 1 and was given false feedback. In the left and center are the results from Experiments 3 and 4, respectively, in which participants were not informed about whether the to-be-learned word, provided as feedback, was true or false. On the right are the results from Experiment 5 in which participants were informed, immediately upon being presented with the to-be-learned word in red, whether that feedback was true or false, and, if false, what the true answer was. Error bars indicate the standard error of the mean

The γ correlations, shown in the left panel of Fig. 4, were computed for Phase 3 of the experiment, in which participants were asked to provide the factually correct answer to each question. When participants had provided the correct answer in the first place and were given true feedback, they were nearly always correct and thus, there was not enough variability to compute γ. When they had been correct in the first place and were given false feedback, the γ correlations were high, in keeping with their original confidence in the correctness of their responses (*M* γ = .72, *SD* = .63, *t*(18) = 4.98, *p* < .001). This result indicates that they knew that the false feedback was false and stuck to their initial answers as being correct. When an error had been committed in Phase 1 and true information was presented as feedback to be learned, the γ correlations were not significantly different from zero in Phase 3 (*M* = .10, *SD* = .45, *t*(23) = 1.04, *p* = .31). Thus, if participants were uncertain about the correctness of the response given as feedback, the delayed hypercorrection effect was not different from zero, a result which echoes that found in Experiment 1. If participants were wrong initially and false feedback was given, there were very few factually correct responses in Phase 3, and only 8 participants had sufficient data for a γ correlation to be computed. It is not surprising, then, that the mean γ was not different from zero (*M* γ = .22, *SD* = .85, *t*(7) = .740, *p* = .483). As with the Phase 2 γ correlations, there were many missing data points, and so the differences among the γ correlations were not significant (*F*(2,36) = 2.49, *p* = .10, ${\eta}_p^2$ = .122, 1 – β = .468).

##### Recall

As indicated in Table 1, when participants were correct in Phase 1 and were given true feedback, they remained correct on the final, Phase 3 test with a high probability (*M* = .99, *SD* = .03). A repeated measures ANOVA was conducted on the remaining three conditions. The main effect of condition was significant (*F*(2, 46) = 89.53, *p* < .001, ${\eta}_p^2$ = .79, 1 – β = 1). Following an error in Phase 1, correct factual recall was significantly worse when participants had been given false feedback (*M* = .02, *SD* = .03) versus true feedback (*M* = .56, *SD* = .14, *t*(23) = 19.57, *p* < .001). When participants had been correct in Phase 1 and were given false feedback, their probability of producing the correct response in Phase 3 was *M* = .69, *SD* = .31. They exhibited better performance in Phase 3 when they had initially been correct and were given false feedback than when they had initially been wrong and received false feedback (*t*(23) = 10.89, *p* < .001). Correct responding in the latter condition was not different from zero. In addition, being correct initially, even when the correct response was followed by false information, resulted in better accuracy on the final test than receiving true feedback following an error (*t*(23) = 2.02, *p* = .055). Finally, recall was higher when an initially correct Phase 1 response was followed by true feedback as opposed to false feedback (*t*(23) = 4.94, *p* < .001).

### Discussion

The hypercorrection results from the first two experiments were further clarified and bolstered by Experiment 3. Participants hypercorrected when they had made an error and were given corrective feedback, but did not hyper'correct' when they had been correct and were given false feedback. When they had been right, it seems plausible that they may have had some knowledge that the feedback they were given was wrong (when it was). Interestingly, though, they also hyper'corrected' when they had made an error in Phase 1 and they were given false feedback following their error. Participants did not appear to be able to recognize misinformation and exclude it from being encoded when they had made an error.

## Experiment 4

A limitation of Experiment 3 was that it was sometimes not possible to compute γ correlations, because it is necessary to have at least one correct and one incorrect response and several different confidence levels in every condition. For this reason, Experiment 4 was a replication with a slight variation. We doubled the number of general information questions (and thus, experimental trials), asking participants 128 questions rather than 64, and increased the number of participants recruited. This experiment frequently took participants several hours to complete. Participants were 40 Columbia University students (30 female and 8 male, plus 2 who did not answer, with an average age of 20.92 years). The participants received a bonus course credit for participating.

### Results

#### Phase 2

##### Hypercorrection

The Phase 2 γ correlations are shown in the center of Fig. 3. In the standard condition, a hypercorrection effect was evident (*M* γ = .32, *SD* = .24, *t*(39) = 8.36, *p* < .001). There was also a hyper'correction' effect when the original answer was incorrect and false feedback was given, both when trials on which participants had received false feedback in Phase 2 and the same erroneous answer that they had given in Phase 1 were included (*M* γ = .12, *SD* = .27, *t*(39) = 2.82, *p* = .008), and when those trials (which occurred with a *M* probability of .029) were removed (*M* γ = .21, *SD* = .25, *t*(39) = 5.30, *p* < .001). When the initial response was correct and false feedback was given, the γ correlation between original confidence and production of the response in red was significantly negative (*M* = −.17, *SD* = .49, *t*(35) = − 2.06, *p* = .047), indicating a confidence-related resistance to remembering the item in red. The γ correlations in the three conditions were significantly different from one another (*F*(2,70) = 19.76, *p* < .001, ${\eta}_p^2$= .36, 1 – β = 1), with the condition in which the original answers were correct followed by false feedback showing lower γ correlations than when the error was followed by correct feedback (*t*(35) = − 2.91, *p* = .006) or by erroneous feedback (*t*(35) = − 2.83, *p* = .008). These two latter conditions both showed γ correlations that were greater than zero and did not differ from one another (*t* < 1).

##### Recall

When participants were correct in Phase 1 and were given true feedback, they were virtually always correct in Phase 2 (*M* = .96, *SD* = .21). A repeated measures ANOVA on the proportion of items recalled in Phase 2 for the three remaining conditions revealed a significant main effect (*F*(2, 78) = 28.98, *p* < .001, ${\eta}_p^2$ = .43, 1 – β = 1). When participants had been correct originally and were given false feedback, recall of the items in red was *.*71, *SD* = .21. When participants had been incorrect originally, recall of the items in red was .53, *SD* = 15 when they were given true feedback and .54, *SD* = .17 when they were given false feedback (*t* < 1). The condition in which the original answers were correct followed by false feedback differed from both of these conditions (*t*(39) = 5.82, *p* < .001, and *t*(39) = 5.70, *p* < .001, respectively).

#### Phase 3

##### Relation of Phase 1 confidence to Phase 3 factual recall

As shown in the center of Fig. 4, when an error had been made in Phase 1 and true feedback was given, a hypercorrection effect emerged in Phase 3 (*M* γ = .10, *SD* = .22, *t*(39) = 2.85, *p* = .007). When an error had been made in Phase 1 and false feedback was given, the γ was not significantly different from zero in Phase 3 (*M* γ = .05, *SD* = .25, *t*(39) = 1.16, *p* > .25). Finally, when participants had provided the correct answer in the first place, were given false feedback, and were then retested for the factually correct answer on the final test, the γ correlations were positive and high (*M* γ = .72, *SD* = .63). Participants recalled the correct answers consistently with their original confidence in the correctness of their Phase 1 responses (*t*(20) = 4.91, *p* < .001). The main effect of condition was significant (*F*(2, 40) = 11.42, *p* < .001, ${\eta}_p^2$= .36, 1 – β = .99).

##### Recall

For Phase 3 accuracy, when participants had been correct originally and were given true feedback, they remained correct, as expected (*M* = .95, *SD* = .12). When participants had initially been correct and received false feedback, their final factually correct performance was .87, *SD* = .22, unlike when they were wrong and received true feedback (*M* = .42, *SD* = .14, *t*(39) = 12.10, *p* < .001). When they had been incorrect originally and were given false feedback, they were nearly always wrong, not surprisingly (*M* = .03, *SD* = .03). However, when participants had been wrong originally but received true feedback, there was a sizable improvement in accuracy on the final test (*t*(39) = 17.89, *p* < .001). Receiving false, relative to true feedback, following a correct response in Phase 1 resulted in a loss of accurate performance on the final test (from .95 to .87, *t*(39) = 2.78, *p* = .008). The false feedback—without any validation of the veracity of that feedback— hurt participants knowledge of what was in fact true.

### Discussion

As had been found in Experiment 3, in this experiment, participants hypercorrected when they had made an error and were given corrective feedback. They also hyper'corrected' when given false information when they had been wrong initially. However, they did not hyper'correct' when they had been correct and were given false feedback.

## Experiment 5

In the final experiment, participants were told directly which answers were correct and which were incorrect, and, if incorrect, what the correct answer was. Otherwise, Experiment 5 was like Experiments 3 and 4. Participants were 25 Columbia University students (7 male and 17 female, plus 1 who did not answer, with an average age of 19.21 years). A course credit was offered in exchange for participating in the experiment. None of the participants had taken part in any of the other experiments in this series. Data from one participant were eliminated because of their low accuracy in Phase 1, which prohibited most analyses from being computed (only 3 out of 64 answers correct, or <5%).

### Results

#### Phase 2

##### Hypercorrection

As shown in the right of Fig. 3, in Phase 2, the mean γ correlation for the standard condition was significantly greater than zero (*M* γ = .39, *SD* = .36, *t*(23) = 5.294, *p* < .001). The γ correlations for false feedback following an incorrect response also showed hypercorrection (*M* γ = .19, *SD* = .38, *t*(23) = 2.457, *p* = .02), although the magnitude of the hypercorrection in these two conditions differed. There was a larger hypercorrection effect when true as opposed to when false feedback was given (*t*(23) = 2.20, *p* = .038). When the questions had originally been answered correctly and false feedback was provided, the γ correlations were not significantly different from zero (*M* = −.01, *SD* = *.*55, *t*(20) < 1).

##### Recall

When participants were correct in Phase 1 and were given true feedback, they were virtually always correct in Phase 2 (*M* = .95, *SD* = .15). A repeated measures ANOVA on the proportion of items recalled in Phase 2 for the three remaining conditions revealed a significant main effect of condition (*F*(2, 46) = 7.74, *p* = .001, ${\eta}_p^2=$ .25, 1 – β = .94). Participants did recall the false feedback, even when they had initially been correct in Phase 1 (*M* = .56, *SD* = .24), although recall of this false information following a correct response initially was lower than recall of the true information following a correct response (*t*(23) = 6.90, *p* < .001). There was no difference in recall of false information following a correct answer versus true feedback following an incorrect response (*t*(23) = 1.00, *p* < .328). However, when participants had been wrong in Phase 1, they remembered the factually true and false feedback differentially in Phase 2 (*t*(23) = 2.79, *p* = .010). The true information was recalled significantly more often than the false information.

#### Phase 3

##### Relation of Phase 1 confidence to Phase 3 factual recall

As shown in the right of Fig. 4, in Phase 3, where the criterion for correctness was being factually correct, the two γ correlations for the conditions in which participants had originally committed an error both showed hypercorrection. Presumably, even though people were tasked with remembering the item in red in the second phase, which could be factually incorrect, the presentation of the true answer, in this experiment—regardless of whether they also had to remember a false answer or not—led to hypercorrection in this third phase. The γ correlations were significantly positive for wrong answers followed by false feedback (*M* = .43, *SD* = .33, *t*(23) = 6.47, *p* < .001) and for wrong answers followed by correct feedback (*M* = .35, *SD* = .36, *t*(24) = 4.73, *p* < .001). The two conditions were not different from one another (*t*(23) < 1). When participants had been correct initially and were given false feedback (and were told that it was false), the Phase 3 γ was positive, .37, *SD* = .71, suggesting that participants were more likely to be correct on the final test when they had had high compared to low confidence in their original responses (*t*(9) = 1.63, *p* = .138).

##### Recall

The most prominent result that emerged as a function of being told which answers were right and wrong was during Phase 3, when people were asked to recall the correct answers. If they had been correct in Phase 1, they almost never made a mistake in Phase 3, despite having been exposed to and remembering the false feedback (*M* = .99, *SD* = .05). Performance in this condition was better than when participants had initially been incorrect and received true feedback (*M* = .63, *SD* = .17, *t*(23) = 10.52, *p* < .001). When they had made a mistake in Phase 1, they also recalled the correct answers better when they had been asked to remember true compared to false feedback (*M* = .55, *SD* = .19, *t*(23) = 2.29, *p* = .032). Likewise, participants showed better Phase 3 performance when they received false feedback following initially correct versus incorrect Phase 1 responses (*t*(23) = 12.03, *p* < .001). Strikingly, there was no difference in final recall performance when participants had been correct originally as a result of whether the to-be-remembered feedback they received was true or false (*t*(23) = 1.23, *p* = .233). Thus, the decrement in final correct performance seen in previous experiments appears to be attributable to participants having been uncertain about the truth of their original responses in the face of false information. When given false information but informed of its truth value, the false information had no effect on final factually correct recall.

## Comparison of Experiment 5 with Experiments 3 and 4

To analyze whether having explicit knowledge about the truth of the feedback impacted γ correlations and the proportion of words correctly recalled in Phases 2 and 3, ANOVAs treating experiment (3 and 4 vs. 5) as a between-participant factor and condition (false feedback following initial correct responses, false feedback following initial incorrect responses, and true feedback following initial errors) as a within-participant factor were conducted.

### Phase 2

#### Hypercorrection

The main effect of experiment, and the interaction between condition and experiment, were not significant (*F* < 1). However, the main effect of condition was significant (*F*(2, 142) = 10.91, *p* < .001, ${\eta}_p^2$ = .13, 1 – β = .99). Being incorrect in Phase 1 and receiving false feedback (*M* = .21, *SD* = .35) resulted in a lower average γ correlation than being incorrect in Phase 1 and receiving true feedback (*M* = .32, *SD* = .34, *t*(87) = −2.20, *p* = .03), but a higher γ correlation than being correct originally but receiving false feedback (*M* = −.06, *SD* = .59, *t*(72) = 3.51, *p* = .001). Wrong answers followed by true feedback (*M* = .32, *SD* = .35) resulted in higher γ correlations than did correct answers followed by false feedback (*t*(73) = 4.39, *p* < .001).

#### Recall

There was a main effect of experiment such that the proportion of correct Phase 2 responses was higher in the experiments in which participants were not informed (3 and 4) than in the experiment in which they were informed (Experiment 5; *F*(1, 86) = 5.94, *p* = .017, ${\eta}_p^2$ = .17, 1 – β = 1). There was also a main effect of condition (*F*(2, 172) = 16.97, *p* < .001, ${\eta}_p^2$ = .12, 1 – β = .92). Being correct in Phase 1 and receiving false feedback (*M* = .66, *SD* = .25) resulted in better recall of the red word in Phase 2 than being incorrect on Phase 1 and receiving either true feedback (*M* = .56, *SD* = .18, *t*(87) = 4.43, *p* < .001) or false feedback (*M* = .53, *SD* = .18, *t*(87) = 5.81, *p* < .001). Being wrong in Phase 1 and receiving true feedback also resulted in significantly better Phase 2 recall of the red word compared to being wrong and receiving false feedback (*t*(87) = 2.25, *p* = .027). These main effects were moderated by a significant interaction between condition and experiment (*F*(2, 172) = 4.98, *p* = .008, ${\eta}_p^2$ = .06, 1 – β = .81). The proportion of correct recall for the red word when false feedback followed a correct response was greater for Experiments 3 and 4 (*M* = .71, *SD* = .25) than for Experiment 5 (*M* = *.*56, *SD* = .25, *t*(86) = 2.47, *p* = .015). Correct recall of the red word when false feedback followed an incorrect response was greater for Experiments 3 and 4 (*M* = .57, *SD* = .17) than for Experiment 5 (*M* = *.*43, *SD* = .19, *t*(86) = 3.29, *p* = .001), but not when true feedback followed an incorrect response (*M* = .57, *SD* = .16 for Experiments 3 and 4 vs. *M* = .56, *SD* = .24 for Experiment 5, *t*(86) < 1).

### Phase 3

#### Relation of Phase 1 confidence to Phase 3 factual recall

The main effect on γ correlations of experiment was not significant (*F*(1, 36) = 2.01, *p* = .165). The main effect of condition was marginally significant (*F*(2, 72) = 3.01, *p* = .056, ${\eta}_p^2$ = .08, 1 – β = .57). Whereas the difference between the γ correlations associated with being incorrect in Phase 1 and receiving false feedback (*M* = .19, *SD* = .41) and being incorrect in Phase 1 and receiving true feedback (*M* = .18, *SD* = .35) was not significant (*t*(71) < 1), being correct and receiving false feedback (*M* = .57, *SD* = .55) resulted in higher γ correlations than both being incorrect and receiving false feedback (*t*(37) = 2.99, *p* = .005) and being incorrect and receiving true feedback (*M* = .14, *SD* = .35, *t*(49) = 4.20, *p* < .001). There was also a significant interaction between condition and experiment (*F*(2, 72) = 3.53, *p* = .034, ${\eta}_p^2$ = .09, 1 – β = .64). The γ correlations for initially incorrect responses followed by true feedback in the uninformed experiments was .35, *SD* = *.*36, and .10, *SD* = .33, for the informed experiment, which was significant (*t*(70) = 3.85, *p* < .001). For initially incorrect responses followed by false feedback, the γ correlations were *M* = .43, *SD* = *.*33, and *M* = .07, *SD* = .40, respectively, which was also significant (*t*(86) = 3.09, *p* = .003). In both cases, the γ correlations were significantly higher in Experiments 3 and 4 (uninformed) than they were in Experiment 5 (informed). There was no difference for initially correct responses followed by false feedback (*t*(48) = −1.19, *p* = .24).

#### Recall

There was an overall effect of experiment such that recall of the factually correct answer in Phase 3 was higher in Experiment 5 than it was in Experiments 3 and 4 (*F*(1, 86) = 120.25, *p* < .001, ${\eta}_p^2$= .58, 1 – β = 1). There was also a main effect of condition (*F*(2, 172) = 235.28, *p* < .001, ${\eta}_p^2$ = .73, 1 – β = 1). Higher accuracy on Phase 3 was seen for correct Phase 1 responses followed by false feedback (*M* = .85, *SD* = .25) relative to incorrect Phase 1 responses followed by both true feedback (*M* = .51, *SD* = .17, *t*(87) = 11.498, *p* < .001) and false feedback (*M* = .17, *SD* = .26, *t*(87) = 22.02, *p* < .001). An error followed by true feedback also produced better performance than an error followed by false feedback (*t*(87) = 14.16, *p* < .001). Finally, there was an interaction between condition and experiment (*F*(2, 172) = 27.07, *p* < .001, ${\eta}_p^2$= .24, 1 –β = 1), such that the proportion of factually correct responses in Phase 3 was higher in every condition in Experiment 5 than in Experiments 3 and 4: initially correct responses followed by false feedback (*M* = .99, *SD* = *.*05 and *M* = .81, *SD* = *.*27, respectively, *t*(86) = 3.25, *p* = .001); initially wrong answers followed by true feedback (*M* = .63, *SD* = *.*17 and *M* = .47, *SD* = *.*15, respectively, *t*(86) = 4.08, *p* < .001); and initially wrong answers followed by false feedback (*M* = .55, *SD* = *.*20 and *M* = .03, *SD* = *.*03, respectively, *t*(86) = 20.65, *p* < .001).

## General discussion

First, these data show that it is not the case that the correction of misinformation is indistinguishable from misinforming people at the expense of previously held correct beliefs. In all five experiments, participants hypercorrected when they had made an error and were given correct feedback to remember. In none of the five experiments did participants hyper'correct' when they had originally been correct and were given false feedback to remember. These results are the first to show empirically that individuals respond differentially, regardless of confidence, as a function of whether they were initially correct or incorrect. They also suggest that people may be able to use second-order knowledge concerning their own metacognitive confidence judgments (Buratti et al., 2013; Dunlosky, Serra, Matvey, & Rawson, 2005).

Second, although these data support the idea that people have some knowledge that differentiates correct and incorrect responses—and results in differential processing of correct responses overwriting errors than for misinformation overwriting correct answers, despite being equally confident in the correctness of these correct and incorrect responses initially—the pattern of the differences was only slightly more exaggerated and clear when people were provided with explicit knowledge of whether they were initially correct or incorrect, and whether the feedback they received in response to their answers was true or false. In the experiments in which participants were given this explicit knowledge (Experiments 2 and 5), compared to those in which such knowledge was not provided (Experiments 1, 3, and 4), similar trends emerged.

Third, when participants had made errors initially, not only did they hypercorrect when given correct answers, but they also hyper'corrected' when they were given false feedback. The fact that participants did not distinguish true and false feedback following an error is remarkable. When people were wrong with high confidence, they were particularly vulnerable to further misinformation.

### The locus of differences in memory for true or false feedback

#### Evaluation of the feedback

An obvious source of the differential processing of feedback following correct answers and errors may be that participants are able to evaluate the truth of the feedback that they were given, and they use this evaluation, along with the additional information that the feedback brings to mind, to bias updating in memory. That telling participants whether the feedback was true or false made almost no difference to their processing suggests that they may have already made this evaluation on their own.

**Fig. 5:**
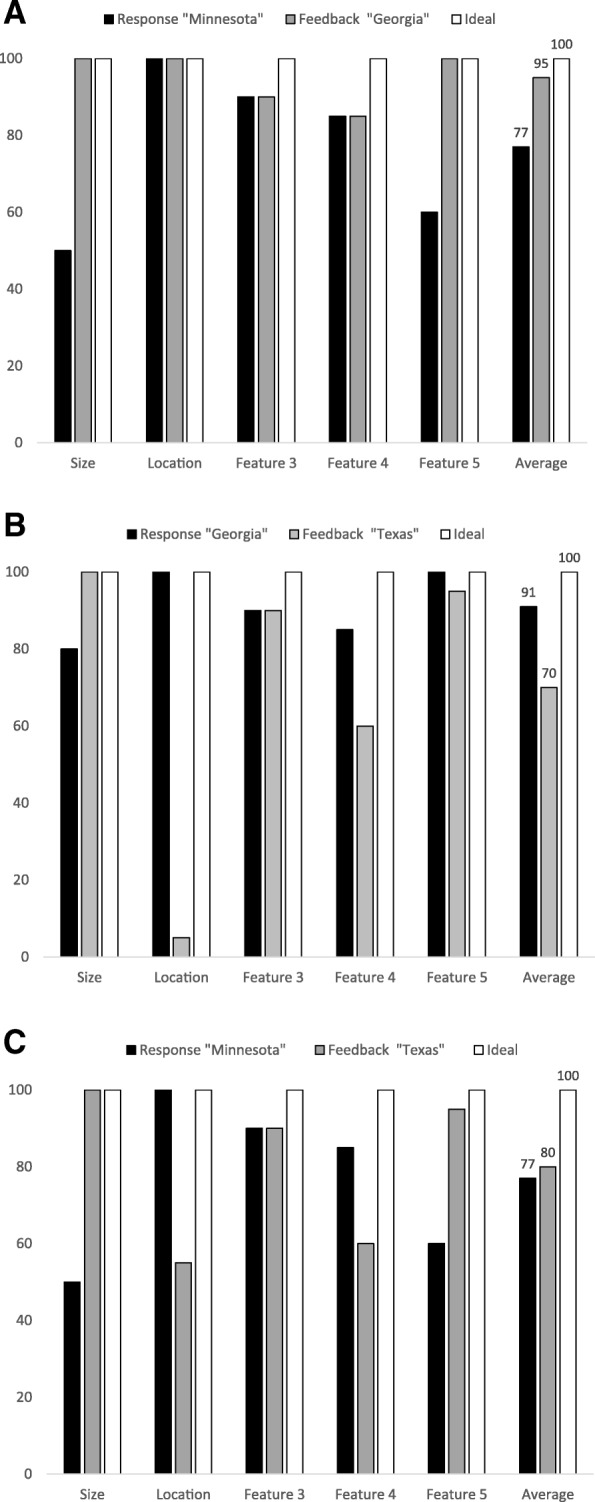
Hypothetical results from a feature-matching model in which the accuracy of relevant features of the response and feedback are compared to guide a decision about whether to adopt or reject the feedback. **a** Incorrect response, true feedback. **b** Correct response, false feedback. **c** Incorrect response, false feedback

A rapid online feature-matching model illustrates this idea, in which memory is updated as a function of the participants’ initial response—correct or incorrect—and the feedback given—true or false (Fig. 5). Here, bars indicate the accuracy of relevant potential features about answers to the question “What is the largest state that’s east of the Mississippi River?” that a participant may use to update memory. In Fig. 5(a), the participant incorrectly responds “Minnesota,” a large state that borders the Mississippi River. However, possible disconfirming information is not considered. The feedback “Georgia” makes this participant realize that Minnesota is too far west. Crucially, this discriminating information (see Gigerenzer, Hoffrage, & Kleinbölting, 1991; Koriat, 2012) is triggered by the feedback, but was not considered during the initial evaluation of their answer to the question. Realizing that they were wrong, they hypercorrect this true feedback.

In Fig. 5(b), the participant is confident that Georgia is one of the biggest states east of the Mississippi, and also they have coded that Georgia is east of the Mississippi. When they receive the false feedback “Texas,” they know immediately that this cannot be a correct response because Texas is west of Louisiana, which borders the Mississippi to the east, and so they do not hyper'correct' to the false feedback.

The last case, illustrated in Fig. 5(c), is potentially the most interesting, from the perspective of understanding why misinformation is adopted. Here, the participant responds “Minnesota” and receives the false feedback “Texas.” If this person was unsure of where the Mississippi River is geographically (which in this hypothetical example is clear, as their initial response was Minnesota, a state that is west of the Mississippi), but does know that Texas is the largest state in the continental U.S., they may hyper'correct'. They realize that their high-confidence response may be wrong and they get no information from the feedback that is disconfirming, and so they are willing to accept the feedback regardless of whether it is true or false and shift their belief towards this false feedback. This interpretation is supported by the fact that in all three of the experiments (3–5) in which false feedback was provided to incorrect responses, participants showed a hyper'correction' effect.

The speed of this postulated evaluative process and the ensuing enhanced processing (for a correct answer following an error) or the inhibition of the enhanced processing (for erroneous feedback following a correct response) is striking. Event-related potential (ERP) evidence indicates that an exaggerated P3a component related to the feedback to high-confidence errors occurs roughly 400 ms post-feedback (Metcalfe et al., 2015). Given this, it is likely that people have some additional knowledge—above and beyond their stated confidence—that they bring to bear even before the feedback is presented.

#### Pre-feedback knowledge

There is considerable evidence that people may have access to other knowledge that is related to their confidence judgments. A number of studies have indicated that a particularly dense semantic landscape is related to highly confident erroneous responses (Eich et al., 2013; Metcalfe & Finn, 2012, 2013; Metcalfe & Miele, 2014; Sitzman & Rhodes, 2010; Sitzman et al., 2015) and that the density of knowledge may facilitate further learning. Butterfield and Mangels (2003) and Butterfield and Metcalfe (2006) provide evidence that people are more familiar with high- than low-confidence questions, as well as with the responses, both correct and incorrect, associated with them—a result which has been borne out by LSA of the relation of the errors to the correct answers (Eich et al., 2012; Metcalfe & Finn, 2012; Metcalfe & Miele, 2014). Metcalfe and Finn (2012, 2013) showed that once people are provided with the correct answer following a highly confident compared to a low-confidence error, they frequently say they “knew it all along.” Thus, even without feedback, participants are more likely to produce the correct answer on a second guess, to choose it on a multiple-choice test, and to guess it when given clues. Given that confidence is related to the density of the semantic landscape, and that the density of the semantic landscape facilitates learning, it is possible that the density of the semantic landscape is responsible for the hypercorrection effect. This view is bolstered by the fact that hyper'correction', in the present experiments, was observed when people were wrong with high confidence and incorrect answers were provided to them to learn. However, presumably the density of the semantic neighborhood increases with confidence in correct (Tunney, 2010) as well as incorrect answers. Thus, this underlying knowledge density factor should facilitate *both* the updating of *correct* as well as *incorrect* responses. However, in the present studies, a hyper'correction' effect was not observed for correct answers, suggesting that some other factor is involved.

It is, of course, possible that participants were disingenuous about their initial confidence ratings, or that there was no equivalency between ratings made for incorrect versus correct responses at the time that the rating was made. In other words, a participant who rates their confidence as 90% and gives the correct response might not mean the same thing as when they give a 90% confidence rating and gives an incorrect response. While this may seem, at first, unlikely, given that confidence ratings were made *before* feedback was given, this possibility is not entirely implausible.

In other paradigms, people sometimes appear to have knowledge that is not captured by simple metacognitive ratings. For example, when people are in a tip-of-the-tongue state they typically express very high feeling-of-knowing ratings (see, Schwartz & Brown, 2014). However, they also sometimes know—and can report if asked—when they are experiencing a blocker that they are certain is wrong (see, Kornell & Metcalfe, 2006). Similarly, Lindsay and Johnson (1989) showed that although people will confidently affirm that they saw a misleading event in one context, if they are pressed more directly about the context of the event, they can reveal that they know that the experience did not occur in the queried context after all. They based their original judgment upon confirmatory fluency information, but can further scrutinize their memory for disconfirming information that alters the simpler assessment. Son (2010) observed that even when participants indicated that they were 100% confident that they had fully learned a particular item, when asked a subsequent question of whether they wanted to restudy the item, they would often opt to do so. Presumably, an item that was fully learned with absolute certainty would not need to be restudied. Thus, in this case, the metacognitive measure indicated that they believed they knew the answer, but their subsequent choices indicated that they knew they did not.

Furthermore, several studies have shown that asking a metacognitive question in different ways can produce different results. Koriat and Bjork (2005) showed that when people were asked to give their confidence ratings about how likely it was that they would remember an item, their subjective expected probability of recall was greater than when the experimenters reframed the question to enquire about how likely it was that they would forget the item. Presumably—while technically asking for the same information—the question about remembering evoked confirmatory evidence while the forgetting question provoked disconfirming evidence, producing different results. Finn (2015) showed that this difference in judgments had consequences. It played out in study choices, wherein people chose to study more in the latter than in the former case. Perhaps, if subjects in the present study had been asked how confident they were that their responses had been false rather than true, the results would have been different. People may have more knowledge about the truth of their answers, even prior to receiving feedback, than a single simple query about confidence taps.

A number of studies have asked people to make responses and confidence judgments multiple times to the same question (Koriat, 2012). These studies follow from the finding that if several people with different viewpoints are asked a question, the answer that the group as a whole produces is more closely aligned with the truth than are the answers from individual participants (Galton, 1907; Wallsten, Budescu, Erev, & Diederich, 1997; Yaniv & Milyavsky, 2007), a phenomenon called the “wisdom of crowds” (Hertwig, 2012). Similarly, if a single individual is tasked with retrieving and making corresponding confidence judgments several times, rather than just once, the answer obtained by combining the different retrieval events can be more accurate than any of the single retrieval events, as Herzog and Hertwig (2014) have demonstrated. Fraundorf and Benjamin (2014) estimated that the advantage gained from the inner crowd is about 1/10 that of using different people, and the effects can sometimes be small (Ariely et al., 2000). Even so, the inner crowd findings demonstrate that people may have untapped knowledge about the accuracy of their retrievals. Additional research is needed to determine whether such additional knowledge might be responsible for the confidence-related differences we observed in the experiments presented here.

Finally, perhaps people’s confidence judgments were systematically inaccurate. Perhaps it really is the case that 90% confidence in one context (for example, after making an error) is not the same as 90% confidence in a different context (for example, after a making a correct response). It is possible that people sometimes impulsively and incorrectly express high confidence, and they are later able to spot when they did so. This possibility could be checked by asking subjects to make metacognitive judgments about the accuracy of their confidence ratings. We were able to find only four studies in which people were asked to make such second-order metacognitive judgments—judgments about their original metacognitive judgments (Buratti et al., 2013; Buratti & Allwood, 2012a, b; Dunlosky et al., 2005). One of these, though—the study by Buratti et al. (2013)—is directly relevant to the present results. In this study, people answered semantic memory questions and gave their confidence about their answers. Later, participants were asked if they wanted to change any of their confidence ratings. Very few ratings were changed. Indeed, so few were altered that the overall accuracy of the relation between confidence and accuracy was unaffected. Even so, when the authors investigated *which* particular ratings were changed, the results were systematic. The confidence ratings associated with errors that had been assigned high-confidence ratings and those associated with correct answers that had been assigned low-confidence ratings were chosen. The changes people made to these ratings were in the direction of greater metacognitive accuracy. These findings favor the idea that people do have some access to the truth of their responses above and beyond what their initial confidence ratings indicate.

It would appear that all of these lines of research bolster the idea that people have some knowledge beyond what their initial metacognitive ratings indicate. It is notable, though, that in all the above cases, people had to be explicitly asked to make the finer-grained judgment or to reassess what they had said before. Only then did the additional knowledge surface. In our experiments, people were never asked to make such a reassessment. If this additional knowledge is responsible for the differences between the patterns of updating following responses that were correct and those that were incorrect, it emerged spontaneously in the experiments described here. Whether this second-order metacognitive knowledge is sufficient to account for our findings, then, remains an empirical question.

In the present experiments, people answered factual questions and were asked, in a straightforward manner, to provide confidence judgments indicating how sure they were that their answers were true. The data presented here provide evidence that people either have access to or gain access to knowledge beyond their original confidence judgments about the correctness of their own retrieval. The present experiments cannot definitively answer whether this knowledge was gained through evaluation of the feedback that was given in response to the initial answers, or whether it was available before the feedback was provided. When this additional information about the truth of their answers emerges, and how it may be put to use, are questions for further empirical research. However, confidence has different memorial consequences, depending on whether the answer was true or false and whether the feedback was true or false. It appears, from the research presented here, that meta-metaknowledge of the truth of an individual’s own answers—knowledge that is not expressed in first-order confidence judgments—differentially impacts cognitive processing and memory.

## Additional file

Additional file 1: List of Stimuli. (DOCX 119 kb)

## Footnotes

1 The term hyper'correction' will be used to refer to enhanced memory for to-be-learned new responses as a function of high compared to low confidence when the new to-be-learned responses are factually incorrect.

2 γ correlations between confidence and recall could sometimes not be computed (if there were no responses in a particular condition, if all responses in a condition were correct or all were incorrect, or if confidence values did not vary, for instance), which accounts for the differences from the expected degrees of freedom in some of the results.

3 Several answers were not in the LSA database, which accounts for the differences in the degrees of freedom.

4 It was not possible to compute γ correlations for the condition in which participants originally generated the correct answer and then were provided with the correct answer, because —not surprisingly—participants nearly always got these answers correct.

5 For example, if participants answered “Orwell” to the question: “What is the last name of the author who wrote *Brave New World*?” they may have also received the incorrect but plausible lure “Orwell” as false feedback.
