# Supplementary material for: Memory and truth: correcting errors with true feedback versus overwriting correct answers with errors
Source: Cogn Res Princ Implic. 2019 Feb 13;4:4. doi: 10.1186/s41235-019-0153-8 (PMC6374496; doi:10.1186/s41235-019-0153-8)
Supplement: Supplementary file 1 — List of Stimuli. (DOCX 119 kb) [file 41235_2019_153_MOESM1_ESM.docx]

**List of Stimuli**

**(Question, followed by the factually correct answer, followed by a plausible, but incorrect answer)**

What is the last name of the second U.S. president?

ADAMS FRANKLIN

What U.S. vice president called liberals 'pampered prodigies' and 'pusillanimous pussyfooters'?

AGNEW NIXON

What U.S. state boasts the most wetlands, with 170 million acres?

ALASKA FLORIDA

What is the capitol of New York?

ALBANY ROCHESTER

What is the longest river in South America?

AMAZON YELLOW

What modern science got started with the 1543 publication of Andreas Vesalius' 'On the Structure of the Human Body'?

ANATOMY PHYSIOLOGY

What is the last name of the actress who received the Best Actress Award for the movie Mary Poppins?

ANDREWS BANCROFT

In which city is the U.S. naval academy located?

ANNAPOLIS NORFOLK

Who was the title character in 'The Merchant of Venice'?

ANTONIO SHYLOCK

What is the first sign of the zodiac?

ARIES AQUARIUS

What U.S. state has two towns named Evening Shade?

ARKANSAS KANSAS

What is the last name of the first person to set foot on the moon?

ARMSTRONG GLENN

What is the name of the island-city believed since antiquity to have sunk into the ocean?

ATLANTIS TROY

In what ancient city were the Hanging Gardens located?

BABYLON ALEXANDRIA

What is the last name of the Spanish explorer who crossed the Isthmus of Panama and found the Pacific Ocean?

BALBOA CORTEZ

What is the name of the lightest wood known?

BALSA BAMBOO

What is the last name of the first man to run the mile in under four minutes?

BANNISTER CUNNINGHAM

What is the last name of the doctor who performed the first successful human heart transplant?

BARNARD DEBAKEY

What is the last name of the judge who was known as the law west of the Pecos?

BEAN PARKER

What was the last name of the female star of the movie Casablanca?

BERGMAN BACALL

What was the crime committed for those dwelling in Dante's lowest level of hell in his work, 'The Inferno'?"

BETRAYAL SLOTH

What is the name of the male star of Casablanca?

BOGART GABLE

What is the last name of Billy the Kid?

BONNEY CODY

Which sport uses the terms gutter and alley?

BOWLING PINBALL

What the last name of the man who was most responsible for photographing the U.S. civil war?

BRADY KODAK

What is the last name of the actor who received the best actor award for the movie On the Waterfront?

BRANDO DEAN

What city is home to the famed Manneke Pis fountain?

BRUSSELS PARIS

What is the name of the poet who originally wrote Don Juan?

BYRON CERVANTES

What Asian nation has the world's largest religious monument?

CAMBODIA CHINA

What is your zodiac sign if you were born on July 15?

CANCER SAGITTARIUS

What is the last name of the criminal known as Scarface?

CAPONE DILLENGER

What animals did the British stop executing for witchcraft in 1712 (use the plural)?

CATS SNAKES

What is the name of the lizard that changes its color to match the surroundings?

CHAMELEON SALAMANDER

What is the last name of the man who wrote Canterbury Tales?

CHAUCER DONNE

What Russian republic was dubbed 'Yeltsin's Vietnam' in 1995?

CHECHNYA AFGHANISTAN

What is the last name of the singer who popularized a dance known as the Twist?

CHECKER BERRY

What is the name of the kind of cat that spoke to Alice in the story Alice's adventures in Wonderland?

CHESHIRE CALICO

In what games are the standard pieces of Staunton design?

CHESS CRIBBAGE

What is the last name of the person who always ended her PBS cooking shows with 'Bon appetit'?

CHILD STEWART

What is the name of the baseball player with the highest lifetime batting average in the major leagues?

COBB RUTH

What is the name of the navigation instrument used at sea to plot position relative to the magnetic north pole?

COMPASS SEXTANT

What is the capitol of Denmark?

COPENHAGEN OSLO

What is the last name of the astronomer who published in 1543 his theory that the earth revolves around the sun?

COPERNICUS GALILEO

What nation's rebels were known as los barbudos, 'the bearded ones'?

CUBA COLOMBIA

What is the last name of the commander who lost the battle of the Little Bighorn River?

CUSTER LEE

What is the unit of sound intensity?

DECIBEL HERTZ

What D-word describes the scattering of the Jewish people around the world?

DIASPORA DISPERSAL

What is the last name of the author who wrote Oliver Twist?

DICKENS HARDY

What is the last name of the actor who portrayed Spartacus?

DOUGLAS HESTON

What contagious virus surfaced in 1977 and came back in 1995 to kill 245 in Zaire?

EBOLA SARS

What is the last name of the man who proposed the theory of relativity?

EINSTEIN HEISENBERG

What's the northernmost Baltic republic?

ESTONIA SWEDEN

Who is known as The Father of Geometry?

EUCLID PYTHAGORAS

What is the name of the unit of measure that refers to a six-foot depth of water?

FATHOM LEAGUE

Where is the home state of the 'Blue Angels'?

FLORIDA CALIFORNIA

What is the last name of the man who supposedly killed Jesse James?

FORD BOOTH

What is the last name of the inventor of the Steamboat Claremont?

FULTON HOWE

What is the last name of the actor who played Rhett Butler in Gone with the Wind?

GABLE COOPER

In what country did voodoo originate?

HAITI CUBA

What college or university, founded in 1636, is the oldest in the United States?

HARVARD COLUMBIA

What is the last name of the boy in the book Treasure Island?

HAWKINS ROBINSON

What is the name of the mountain range in which Mt. Everest is located?

HIMALAYAS URALS

What is the last name of the 1979 Oscar winner who thanked his parents for not practiing birth control?

HOFFMAN HANKS

What did Robert Redford steal and resell for pocket money as a teenager? (use the plural)

HUBCAPS WATCHES

What is the name of an inability to sleep?

INSOMNIA APNEA

What was the first nation to picture Albert Einstein on banknotes?

ISRAEL GERMANY

What is the official language of the Most Serene Republic of San Marino?

ITALIAN SWAHILI

In the bible, whose name was changed to Israel?

JACOB ABRAHAM

What is the only property an orthodox Hindu woman can own?

JEWELRY COW

What is the last name of the European author who wrote The Trial?

KAFKA MALRAUX

What is the last name of the person who defined 'The Beat Generation'?

KERUOAC GINSBERG

What is the last name of the man who wrote the Star Spangled Banner?

KEY ROSS

What is the last name of the man who created the comic strip Woody Woodpecker?

LANTZ SCHULZ

What is the last name of the player who temporarily ended his career of 12 years with an NHL-high .823 goals-per-game average?

LEMIEUX LEBECQUE

What is the last name of the tennis champ who held the men's number one spot for a record 270 weeks?

LENDL CONNORS

What country's capital is Tripoli?

LIBYA ALGERIA

What did Armand Fizeau measure the speed of in 1849?

LIGHT SOUND

What European city hosted the last and biggest world's fair of the 20th century?

LISBON OSLO

What is the last name of the man who began the reformation in Germany?

LUTHER CALVIN

What is the last name of the person who tested positive for banned decongestants, scuttling Argentina's chances at the 1994 World Cup?

MARADONA PELE

What is the Taj Majal made of?

MARBLE GRANITE

What is the last name of the union general who defeated the Confederate army at the Civil war battle of Gettysburg?

MEADE GRANT

What city was Martin Luther King Jr. assassinated in?

MEMPHIS MONTGOMERY

What country is the new Volkswagen Beetle produced in?

MEXICO GERMANY

Which dynasty was in power throughout the 1500's in China?

MING HAN

What was the name of the Union Ironclad ship that fought the Confederate ironclad Merrimack?

MONITOR LIBERTY

What is the name of the villainous people who lived underground in H. G. Well's book The Time Machine?

MORLOCKS BROBDIGNAGIANS

What is the last name of the composer who wrote the opera Don Giovanni?

MOZART PUCCINI

What is the name of the submarine in Jule Vernes's 20000 Leagues under the Sea?

NAUTILUS PEQUOD

What is the most populous country in Africa?

NIGERIA EGYPT

From what plant does natural vanilla flavoring come?

ORCHID LILY

What is the last name of the man who assassinated President John Kennedy?

OSWALD SIRHAN

What is the capitol of Canada?

OTTAWA TORONTO

What is the name of the largest ocean on earth?

PACIFIC ARCTIC

What country lost wars to India in 1947, 1965, and 1971?

PAKISTAN ENGLAND

What is the last name of the entertainer who claims she's not offended by dumb blonde jokes because she's 'not dumb' and 'not blonde'?

PARTON MIDLER

What is the name of the mythical bird that destroys itself on a burning altar so that a new bird can emerge from the ashes?

PHOENIX ALBATROSS

What is the name of the process by which plants make their food?

PHOTOSYNTHESIS OSMOSIS

What is the name of Socrates' most famous student?

PLATO ARISTOTLE

What Italian city was destroyed when Mount Vesuvius erupted in 79 A.D.?

POMPEII SIENNA

What country's ships transported four million African slaves to Brazil?

PORTUGAL SPAIN

What is the last name of the first flier to fly solo around the world?

POST WRIGHT

In addition to the Kentucky Derby and the Belmont Stakes what horse race comprises the Triple Crown?

PREAKNESS LEXINGTON

What was Cinderella's coach made from?

PUMPKIN GOLD

What is the name of a dried grape?

RAISIN CURRANT

What is the last name of the husband-wife spies who were electrocuted in 1951 for passing atomic secrets to Russia?

ROSENBERG HESS

What was the last name of the woman who supposedly designed and sewed the first American flag?

ROSS ADAMS

What movie has Steve Martin noting, 'It's not the size of a nose that matters, it's what's in it'?

ROXANNE JERK

What is the last name of the baseball player who had the most home runs in a single season prior to 1961?

RUTH MANTLE

What is the last name of the doctor who first developed a vaccine against polio?

SALK FLEMING

What is the name of the author who received the Pulitzer Prize for his writings about Abraham Lincoln?

SANDBURG WESTON

What is the name of the navigation instrument used at sea to plot position by the stars?

SEXTANT ASTROLABE

What is the name of the brightest star in the sky excluding the sun?

SIRIUS ANTARES

What country's civil war was described as a 'rehearsal for World War II'?

SPAIN POLAND

What was the name of the mythological creature with the head of a woman and the body of a lion that killed all those unable to solve its riddle?

SPHINX CHARYBIDIS

According to the FDA, what's the only fish that produces real caviar?

STURGEON SALMON

What movie character was Elmo Lincoln the first to portray?

TARZAN SPIDERMAN

Which sport is associated with Wimbleton?

TENNIS HORSERACING

What is the name of the river that runs through Rome?

TIBER ETNA

In what country is the Dalai Lama's palace?

TIBET INDIA

What is the last name of the Russian novelist Teddy Roosevelt called 'a sexual and moral pervert'?

TOLSTOY NABOKOV

What is the name of the crime in which a person purposely betrays his country?

TREASON SEDITION

What's heaven to fallen Norse warriors?

VALHALLA ORION

In which country is Angel Falls located?

VENEZUELA CANADA

What drug so impressed financier Alan Greenberg that he gave $1 million in 1998 to disseminate it to the poor?

VIAGRA PROZAC

What is the last name of the artist who is famous for producing images of Campbell's soup cans?

WARHOL POLLACK

What was the name of Alexander Graham Bell's assistant?

WATSON HARVEY

What is the last name of the person who invented the cotton gin?

WHITNEY FRICK
